# 2077. Utility of a Real-Time Spatiotemporal Mapping Surveillance System in Detection of Healthcare-Associated Acute Respiratory Viral Infection Clusters in a Tertiary Healthcare Institution

**DOI:** 10.1093/ofid/ofad500.147

**Published:** 2023-11-27

**Authors:** Indumathi Venkatachalam, Edwin Philip, Jean XY Sim, Weien Chow, Yiying Cai, Nicholas Graves, Sean Whiteley, Shalvi Arora, Maybelle Auw, Daniel Chuanwen Tiang, Siow Leng Neo, Joseph Kin Meng Cheong, Wei Wei Hong

**Affiliations:** Singapore General Hospital, Singapore, Not Applicable, Singapore; Singapore General Hospital, Singapore, Not Applicable, Singapore; Singapore General Hospital, Singapore, Not Applicable, Singapore; Changi General Hospital, Singapore, Not Applicable, Singapore; Duke NUS Medical School, Singapore, Singapore; Duke-NUS Medical School, Singapore, Not Applicable, Singapore; Axomem, Singapore, Not Applicable, Singapore; Singapore General Hospital, Singapore, Not Applicable, Singapore; Axomem, Singapore, Not Applicable, Singapore; Singhealth, Singapore, Not Applicable, Singapore; Singhealth, Singapore, Not Applicable, Singapore; Singapore General Hospital, Singapore, Not Applicable, Singapore; SingHealth, Singapore, Not Applicable, Singapore

## Abstract

**Background:**

Transition to endemic COVID-19 has been associated with a rise in community respiratory viral infections (ARIs) with a corresponding increase in healthcare-associated ARIs (HA-ARIs) (Figure 1). 4D-Disease Outbreak Surveillance System (4D-DOSS) is a real-time spatiotemporal mapping surveillance system being developed to detect healthcare-associated infection clusters. We aimed to assess 4D-DOSS’s utility in detection of HA-ARI clusters.

**Figure:**
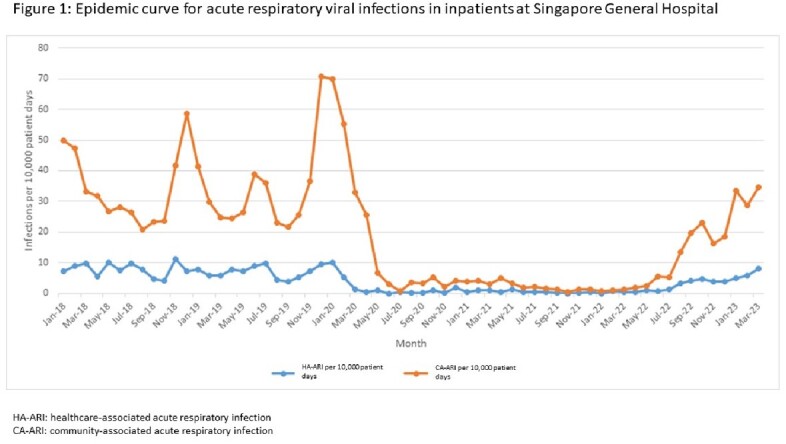

**Methods:**

4D-DOSS is a system that integrates and maps clinical, laboratory and patient movement data onto a digital twin of the hospital’s physical space. In addition to a virtual mapping replica of Singapore General Hospital, a 2000-bedded tertiary hospital in Singapore, it constitutes detailed healthcare cloud architecture and surveillance algorithms.

Respiratory specimens from inpatients with ARI symptoms are tested for 16 human respiratory viral pathogens via a respiratory virus multiplex PCR (RV16) panel. HA-ARI constitutes first positive sample beyond the maximum incubation period of the corresponding virus (from admission date). Earlier positive test is categorized as community-associated ARI (CA-ARI). Two or more patients with spatial temporal overlap of three-days or less, during the infectious period (7-days) of an index patient are deemed a HA-ARI cluster.

**Results:**

Incidence of HA-ARI, as per 10,000 patient-days was 7.4 pre-COVID-19 pandemic (Jan 2018 to Dec 2019), 1.5 during pandemic (Jan 2020 to Dec 2022) and 6.4 during transition to endemicity (Jan 2023 – April 2023). Between September 2018 and December 2018, one influenza cluster of ten inpatients (Nov 2018) was identified in the proof-of-concept version of 4D-DOSS. There were four HA-ARI clusters during the Jan 2020-Dec 2022 COVID-19 pandemic phase. 19 HA-ARI clusters were identified between Jan 2023-April 2023.

**Conclusion:**

4D-DOSS can detect HA-ARI clusters and has potential to trigger an alert-response process for more effective infection prevention. It enables study of infectious disease transmission kinetics in real-time.

**Disclosures:**

**Sean Whiteley, BSc (IT)**, Axomem: Board Member|Axomem: Fees received for service and software licenses **Maybelle Auw, MBBS**, Axomem: Work for company that received fees for service and licensing

